# A Novel Ocean Thermal Energy Driven System for Sustainable Power and Fresh Water Supply

**DOI:** 10.3390/membranes12020160

**Published:** 2022-01-28

**Authors:** Qingfen Ma, Yun Zheng, Hui Lu, Jingru Li, Shenghui Wang, Chengpeng Wang, Zhongye Wu, Yijun Shen, Xuejin Liu

**Affiliations:** 1 College of Mechanical and Electrical Engineering, Hainan University, Haikou 570228, China; zyunzoey@163.com (Y.Z.); ljr@hainanu.edu.cn (J.L.); wuzhongye@126.com (Z.W.); lxj2669@163.com (X.L.); 2 Institute of Environment and Plant Protection, Chinese Academy of Tropical Agriculture Sciences, Haikou 571101, China; aaaluhui@163.com; 3 The Institute of Seawater Desalination & Multipurpose Utilization, MNR, No.55, Hanghai Road, Nankai District, Tianjin 300192, China; cpwang2003@foxmail.com; 4 State Key Laboratory of Marine Resources Utilization in South China Sea, Haikou 570228, China

**Keywords:** OTEC, organic rankine cycle, membrane distillation, thermal efficiency

## Abstract

The ocean thermal energy conversion (OTEC) is a potential substitute for traditional power plants in tropical islands and coastal regions. However, the OTEC power generation cycle has low thermal efficiency and the integrated utilization is imperative, in which an OTEC coupled with seawater desalination is the most attractive option. Membrane distillation (MD) has distinct advantages making itself a competitive process for seawater desalination, especially the feature that the drained warm seawater from the OTEC power plant can be recycled, improving the integrated output of the OTEC system. In this study, an innovative OTEC system coupling a power generation sub-cycle (PGC) and a water production sub-cycle (WPC) was proposed, composed of the upstream organic Rankine cycle and the downstream membrane distillation modules. The mass, energy and exergy balance of the individual equipment, the sub-cycles and the whole system were performed by constructing the corresponding balance models. The thermal dynamic parameters were calculated, and the performance of power generation and water production was predicted. The results showed that by coupling with the MD desalination, the thermal efficiency of the OTEC system can be greatly improved from 2.19% to 25.38% while the exergy efficiency changed little. For a 100 kW OTEC power generation cycle, the water production rate approached 58.874 t/d. In addition, the economic analysis based on the electricity and water sale was carried out, and the profit can be improved by extra water production, especially in the Hawaii and Rainbow Beach by nearly 20%.

## 1. Introduction

The ocean contains abundant renewable resources, with the main forms of utilization including tidal energy, wave energy and ocean thermal energy (OTE). Ocean thermal energy conversion (OTEC) involves the development of thermal energy caused by the temperature difference between the surface water and deep seawater which should be above 18 °C for effective conversion. As one of the ocean energies, OTEC is renewable and emits no pollutant to the environment, and it has more advantages compared to other ocean energies. It absorbs far more solar energy than the global total energy consumption, indicating its enormous reserves; the energy output is stable and hardly changed with the day or night, alleviating the influence of fluctuation or intermittence on the grid; the conversion forms are various, not limited to power generation, and especially suitable for a tropical island with energy, food and water shortages.

OTEC is a potential substitute for traditional power plants, which can provide continuous power production throughout the year. At the beginning of the 20th century, the first open-cycle experimental power station of OTEC with an output of 22 kW was built in Cuba [1], marking the start of the experimental stage of OTEC research. In the 1970s, several OTEC experimental power stations were established in Saga University, Japan and Hawaii University, USA, respectively [2]. By the 1980s, Japan became the leader in the research field of OTEC technology [3], and the power generated by the experimental power stations was up to 100 kW. After the 1990s, the installed capacity of OTEC plants increased rapidly. The United States built a 210 kW shore-based open-cycle power station in Hawaii, and realized the joint supply of electric power and freshwater [4]. In recent years, led by the United States and France, the research and development of 10 MW commercial equipment has been carried out, and OTEC application has been gradually promoted and commercialized. Since the temperature difference of the OTEC system is much smaller than that of the traditional thermoelectric or nuclear power system, the thermal efficiency of the cycle is much lower, usually 2% to 4% [5]. Therefore, the research on the OTEC power generation system mainly focused on the improvement of thermal efficiency, such as the screening of working fluid [6], system optimization [7] and performance improvement of key equipment [8].

Although the OTE is a green renewable energy, it cannot compete with the traditional power supply due to the low thermal efficiency and high fixed costs. Thus, the integrated utilization of OTEC is imperative to improve the thermal efficiency and economics of OTEC system, speeding up the commercialization. Meanwhile, there is a bright future for its integrated utilization on the island, especially the tropical island. Up to now, there are several integrated utilization options, including: (1) seawater desalination treatment [9]; (2) hydrogen and ammonia production [10]; (3) refrigeration [11], and so on.

Among above OTEC integrated utilization options, the combination of power generation and seawater desalination is the most attractive, since the system can meet the power and fresh water demand at the same time, which are both urgent problems faced by islands. Magesh [12] proposed an integrated OTEC power and a desalination plant. For every 1 MW of power generation, nearly 2.28 million liters of portable water can be obtained per day to alleviate the water shortage crisis in arid areas. Kim et al. [13] equipped an OTEC system with multiple condensers to flexibly adjust the power and water production rate and optimize the OTEC cycle. They also predicted that using the vacuum membrane distillation instead of the flash evaporation in the seawater evaporator could effectively reduce the evaporator volume. Park et al. [9] combined the OTEC system with the solar system to produce electricity and desalinate water simultaneously. Compared with the pure OTEC system, the working fluid was heated by the solar collector to increase the temperature difference of the power cycle. As a result, the efficiency of the system reached 3.9 times higher than that of the pure OTEC power plant. Soto and Vergara [14] reported an integrated utilization system of an ammonia Rankine cycle and seawater desalination and constructed a thermodynamic model to evaluate the system efficiency, the freshwater production and the power generation increase. Zhou et al. [15] proposed a hybrid OTEC system combining cooling, desalination and power generation (CCDP) together for remote islands, and evaluated the system performance according to the energy, exergy and economy analysis. The energy saving rate and thermal efficiency reached 33.72% and 29.33%, respectively.

The coupled seawater desalination process in OTEC integrated utilization is usually low-pressure flash evaporation for its lower operating temperature. However, it is not conducive to the offshore applications due to the large volume of key equipment and high vacuum degree. Multi-stage flash (MSF), low-temperature multi-effect distillation (MED) and other thermal methods need a higher heat source temperature than that of the OTEC, so additional heat sources such as solar and geothermal energy are required for auxiliary heating, which depends on the local conditions and is not universal. Reverse osmosis (RO) is the most widely used commercial desalination process, but it requires a power supply, which conflicts with power generation.

Membrane distillation (MD) is a membrane separation process with hydrophobic porous membrane as isolation medium and steam pressure difference on both sides of membrane as a driving force of mass transfer [16]. After vaporizing on the membrane surface, volatile components on the feed side pass through the membrane pore in the form of steam, which is condensed and collected on the permeate side to separate the mixture. As one of the most promising water treatment technologies, it has distinct advantages compared with other separation technologies, such as lower operating temperature [17], higher rejection rate [18], higher fouling resistance and lower operating pressure [19] and is suitable for the utilization of low-grade heat.

According to the characteristics of OTEC and MD mentioned above, they have great coupling potential to generate power and fresh water simultaneously for tropical islands. Besides, by using MD instead of the low-pressure flash process, the volume of seawater evaporator can be greatly reduced by more than 90% [20], which can reduce the manufacturing, transportation and maintenance cost of the equipment, facilitate the OTEC application in offshore platform where the space limitation must be considered, and be helpful for the remote tropical island to reach targets of energy self-sufficiency and transition to 100% sustainable energy systems. However, the research on the coupling of the OTEC and MD was rarely reported up to now and it is urgent to investigate its feasibility.

In this paper, to investigate the coupling potential of the OTEC and MD for the sustainable power and water supply of the remote tropical islands and offshore platform, we propose a novel OTEC-DCMD (Direct contact membrane distillation) integrated system. A DCMD process is embedded after the OTEC power cycle, to improve the energy utilization efficiency by fully utilizing the drained warm seawater from the OTEC power cycle.

The simple organic Rankine cycle is adopted for the OTEC power generation with the least equipment requirement and cost. The DCMD instead of low-pressure flash for seawater desalination results in compact and space-saving equipment. Appling the detailed mathematical model of the integrated system, the mass, energy and exergy balance of the individual equipment, the sub-cycles and the whole system are performed. The performances of power generation and water production are analyzed by thermal calculations, the constructed CFD (Computational Fluid Dynamics) model and sale profit prediction, based on which the feasibility of the integrated systems is investigated. The structure of this paper is as follows:

1. In this paper, we proposed a novel ocean thermal energy driven system for sustainable power and fresh water supply. The integrated system consisting of organic Rankine cycle and DCMD desalination will be introduced in Section 2.
2. A detailed mathematical model of the proposed cycle is to be established from the perspectives of thermodynamics and transmembrane transmit in Section 3.
3. Thermodynamic analyses on the proposed system will be carried out and the output performance of the system will be discussed in Section 4.
4. The conclusions of this work will be given in Section 5.

## 2. Description of the Integrated OTEC-DCMD System

**Figure 1:**
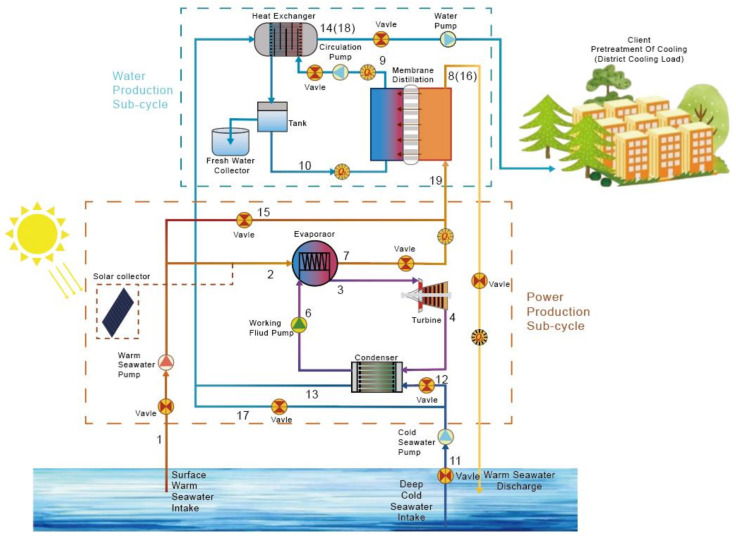
Schematic illustration of the proposed integrated the ocean thermal energy conversion (OTEC) system.

The system proposed in this paper is an integrated OTEC system for both power generation and water production. Residual thermal and cooling energy drained from the OTEC power generation cycle are used to obtain freshwater, improving the energy efficiency of OTEC and meeting the power and water demand for tropical island simultaneously. The composition of the proposed integrated OTEC system is shown in Figure 1. The total system is divided into two sub-cycles, the power generation sub-cycle (PGC) and water production sub-cycle (WPC).

### 2.1. Power Generation Sub-Cycle (PGS)

The PGC is an organic Rankine cycle with ammonia as its working fluid, composed of an evaporator, a condenser, a steam turbine and a pump. The seawater from both the ocean surface and a certain depth are adopted as the heat source and sink, respectively. The warm surface water 1 is pumped into the evaporator by the warm seawater pump, and the high-pressure saturated ammonia 6 flows into the evaporator through the working fluid pump, absorbing the heat from warm seawater and vaporizing into the high-pressure ammonia steam 3, then passes through the turbine with the enthalpy drop converted into work. The low-pressure exhausted ammonia steam 4 is cooled by the deep cold seawater through the condenser, converted into liquid ammonia 5 and then returned to the working fluid pump to restart the cycle. Due to the seasonal changes, the temperature of the surface seawater may vary. In order to ensure that the OTEC turbine can obtain a stable inlet temperature and pressure, a solar collector is introduced for temperature compensation, the required heat collection volume is small and has little effect on the overall equipment volume.

### 2.2. Water Production Sub-Cycle (WPC)

In the WPC, the warm seawater discharged from the PGC 7 enters the feed channel of the direct contacting membrane distillation (DCMD) modules. If the flow rate of the discharged warm seawater is not enough to produce the demanded fresh water, additional surface seawater can supplement the feed channel though the bypass 15. The cold seawater discharged from the condenser in the PGC 13 is used to cool the fresh water in the permeate channel through a heat exchanger. Thus, the fresh water cycled in the permeate channel can maintain a constant temperature. If the flow rate of the discharged cold seawater is not fast enough for cooling, additional deep seawater can supplement the feed channel though the bypass 17. Due to the steam pressure difference between the feed and the permeate channel, the water vapor is transferred from the warm channel to the cold channel through the holes of the hydrophobic breathable membrane and condenses into fresh water 9. The cold seawater passing through the heat exchanger 13 can be discharged or also be exported as the precooling of the air conditioning system and the seawater from the feed channel is discharged directly.

## 3. Mathematical Modelling of Mass, Energy and Exergy Balance

In this section, the proposed integrated OTEC system is analyzed on the basis of mass, momentum and energy transfer laws. The thermodynamic parameters of the PGC are obtained from the simulating results of the Aspen plus software. The water production parameters are predicted through the simulation of the cross-membrane flow in the DCMD module by the CFD commercial software. Based on the above results, the mass, energy and exergy analysis of each unit and the whole system are carried out.

To simplify the analysis, some necessary and reasonable assumptions are made as follows:

1. Ignore the heat loss in the system.
2. The system is in the steady-state operation.
3. All liquids are non-compressible and have uniform speed.
4. The membrane has good hydrophobic and air permeability, regardless of membrane wetting.
5. Ignore the kinetic and potential energy variation of fluid flowing between equipment.

### 3.1. Balance Equation of the Integrated System

Thermodynamic analysis is performed based on the mass, energy, entropy and exergy balance equations. The mass is conserved throughout the system [21], and Equation (1) represents that the mass flow into the system is equal to the mass flow out.

(1) $$\sum_{}^{}{\dot{m}}_{\mathrm{in}}=\sum {\dot{m}}_{\mathrm{out}}$$

According to the first law of thermodynamics, the energy conservation equation of the system is obtained as shown in Equation (2). The left side and the right side represent the energy of the output and input system respectively [22].

(2) $${\dot{Q}}_{\mathrm{in}}+{\dot{W}}_{\mathrm{in}}+{\sum {\dot{m}}_{\mathrm{in}}{\left(h\right)}_{\mathrm{in}}={\dot{Q}}_{\mathrm{out}}+{\dot{W}}_{\mathrm{out}}+\sum \dot{m}}_{\mathrm{out}}{\left(h\right)}_{\mathrm{out}}$$

There are several types of energy in the input and output systems: *W* (kW), *Q* (kW) and *h* (kW) denote the work, heat flow rate and enthalpy carried by the working fluid.

Equation (3) represents the entropy balance of the total system [23].

(3) $$\sum {\dot{m}}_{\mathrm{in}}s_{\mathrm{in}}+\sum {\left(\frac{\dot{Q}}{T}\right)}_{\mathrm{in}}+{\dot{S}}_{\mathrm{gen}}=\sum {\dot{m}}_{\mathrm{out}}s_{\mathrm{out}}+\sum (\frac{\dot{Q}}{T}{)}_{\mathrm{out}}$$

The left and right sides of Equation (4) represent the input and output of exergy.

(4) $$\sum {\dot{m}}_{\mathrm{in}}ex_{\mathrm{in}}+\sum \dot{E}x_{{\;\dot{Q}}_{\mathrm{in}}}+\sum \dot{E}x_{{\;\dot{W}}_{\mathrm{in}}}=\sum {\dot{m}}_{\mathrm{out}}ex_{\mathrm{out}}+\sum \dot{E}x_{{\dot{Q}}_{out}}+\sum \dot{E}x_{{\;\dot{W}}_{\mathrm{out}}}+\dot{E}x_{\mathrm{dest}}$$

where *Ex*_Q_, *Ex*_W_ and *Ex*_dest_ denote the exergy rates of heat transfer and work, and exergy destruction. “ex” characterizes the specific exergy, composed of the physical, chemical, potential and kinetic exergy and can be approximately expressed as:

(5) $$ex\approx ex_{\mathrm{ph}}=h-h_{0}-T_{0}(s-s_{0})$$

The heat exergy and work exergy can be calculated by Equations (6) and (7), respectively.

(6) $$\dot{E}x_{Q}=(1-\frac{T_{0}}{T})\dot{Q}$$

(7) $$\dot{E}x_{w}=\dot{W}$$

### 3.2. Balance Equation of Power Generation Sub-Cycle

The PGC consists of an evaporator, a condenser, a turbine and a working fluid pump. Based on the steady-state operation assumption, the thermodynamic analysis of each component is carried out.

For the evaporator, the balance equations are as follows:

(8) $$\mathrm{Mass}:\;{\dot{m}}_{2}={\dot{m}}_{7};{\dot{m}}_{3}={\dot{m}}_{6}$$

(9) $$\mathrm{Energy}:\;{\dot{m}}_{2}h_{2}+{\dot{m}}_{6}h_{6}={\dot{m}}_{7}h_{7}+{\dot{m}}_{3}h_{3}$$

(10) $$\mathrm{Entropy}:\;{\dot{m}}_{2}s_{2}+{\dot{m}}_{6}s_{6}+{\dot{S}}_{\mathrm{gen},\;\mathrm{Eva}}={\dot{m}}_{7}s_{7}+{\dot{m}}_{3}s_{3}$$

(11) $$\mathrm{Exergy}:\;{\dot{m}}_{2}ex_{2}+{\dot{m}}_{6}ex_{6}={\dot{m}}_{7}ex_{7}+{\dot{m}}_{3}ex_{3}+Ex_{\mathrm{des},\;\mathrm{Eva}}$$

For the turbine, the balance equations are as follows:

(12) $$\mathrm{Mass}:\;{\dot{m}}_{3}={\dot{m}}_{4}$$

(13) $$\mathrm{Energy}:\;{\dot{m}}_{3}h_{3}={\dot{m}}_{4}h_{4}+{\dot{W}}_{t}$$

Ignoring the potential energy changes of import and export, *W*_t_ ≈ *W*_s_, where *W*_s_ is shaft work.

(14) $$\mathrm{Entropy}:\;{\dot{m}}_{3}s_{3}+{\dot{S}}_{\mathrm{gen},t}={\dot{m}}_{4}s_{4}$$

(15) $$\mathrm{Exergy}:\;{\dot{m}}_{3}ex_{3}={\dot{m}}_{4}ex_{4}+{\dot{W}}_{t}+Ex_{\mathrm{des},t}$$

For the condenser, the balance equations are as follows:

(16) $$\mathrm{Mass}:\;{\dot{m}}_{4}={\dot{m}}_{5};\;{\dot{m}}_{12}={\dot{m}}_{13}$$

(17) $$\mathrm{Energy}:\;{\dot{m}}_{4}h_{4}+{\dot{m}}_{12}h_{12}={\dot{m}}_{5}h_{5}+{\dot{m}}_{13}h_{13}$$

(18) $$\mathrm{Entropy}:\;{\dot{m}}_{4}s_{4}+{\dot{m}}_{12}s_{12}+{\dot{S}}_{\mathrm{gen},\mathrm{con}}={\dot{m}}_{5}s_{5}+{\dot{m}}_{13}s_{13}$$

(19) $$\mathrm{Exergy}:\;{\dot{m}}_{4}ex_{4}+{\dot{m}}_{12}ex_{12}={\dot{m}}_{5}ex_{5}+{\dot{m}}_{13}ex_{13}+Ex_{\mathrm{des},\mathrm{con}}$$

For the working fluid pump, the balance equations are as follows:

(20) $$\mathrm{Mass}:\;{\dot{m}}_{5}={\dot{m}}_{6}$$

(21) $$\mathrm{Energy}:\;{\dot{m}}_{5}h_{5}+{\dot{P}}_{\mathrm{wf}}={\dot{m}}_{6}h_{6}$$

(22) $$\mathrm{Entropy}:\;{\dot{m}}_{5}s_{5}+{\dot{S}}_{\mathrm{gen},\mathrm{WF}}={\dot{m}}_{6}s_{6}$$

(23) $$\mathrm{Exergy}:\;{\dot{m}}_{5}ex_{5}+{\dot{P}}_{\mathrm{WF}}={\dot{m}}_{6}ex_{6}+Ex_{\mathrm{des},\mathrm{WF}}$$

For the PGC, the absorbed heat from the heat source can be calculated by Equation (24).

(24) $$Q_{\mathrm{in}}={\dot{m}}_{\mathrm{WF}},h_{3}-h_{5},$$

The output power of the turbine *W*_t_ can be obtained by the Equation (25).

(25) $$W_{t}=\tau {\dot{m}}_{\mathrm{WF}}(h_{3}-h_{4})$$

where *τ* is the efficiency of the turbine.

The power consumption of the working fluid pump *P*_WF_ can be obtained from Equation (26).

(26) $$P_{\mathrm{WF}}=\frac{V_{\mathrm{WF}}(p_{6}-p_{5})}{\theta_{\mathrm{WF}}}$$

where *V*_WF_ is volume flow rate of the working fluid (m^3^/s), *p*_5_ and *p*_6_ the pressure at working fluid pump inlet and outlet (kPa), and *θ*_WF_ the working fluid pump efficiency.

The net output power of the PGC is:

(27) $$W_{\mathrm{out}}=W_{t}-P_{\mathrm{WF}}$$

### 3.3. DCMD Water Production Sub-Cycle

#### 3.3.1. Mass and Heat Transfer Model

By means of CFD simulation, the mass and heat transfer model of DCMD desalination are constructed by considering both the flow behavior and heat transfer in the flow channels of the DCMD module and through the membrane pores.

(1) Macro-flow and heat transfer in the feed and permeate channel

The macro-flow in the feed and permeate channel are simulated based on the continuity equation, N-S equations, the energy equation and the *k*-*ε* turbulence model.

(2) Micro flow of the vapor molecules and the heat transfer across the membrane

The micro transmembrane flow of water vapor causes the mass transfer between the feed and the permeate channel. During the transmembrane mass transfer process of DCMD, the membrane mass flux (mass flow rate per unit membrane area) can be calculated by Equation (28).

(28) $$J_{M}=K_{M}\left(p_{F,W}-p_{P,W}\right)$$

where *K*_M_ (kg·m^−2^·s^−1^·Pa^−1^) is the mass transfer coefficient and depends on the transmembrane mass transfer mode. *p*_F,W_ (Pa) and *p*_p,w_ (Pa) are the partial pressures of water vapor on the feed channel and the permeate channel, respectively. The subscripts “F” and “P” represent the feed channel and the permeate channel, respectively, and the subscripts “W” and “M” represent the parameters of main flow and on the membrane surface. *p*_F,W_ can be calculated by Equation (29), where *γ*_W_ is the activity coefficient of water, which is calculated by Equation (30) [24]; *X*_NaCl_ is the molar fraction of NaCl in feed seawater and is calculated by Equation (31).The saturated vapor pressure *p*_v_^s^(*T*) (Pa) of pure water at different temperatures *T* (K) can be obtained from the Antoine Equation (32).

(29) $$p_{F,W}=\left(1-X_{\mathrm{NaCl}}\right)p_{V}{}^{S}\left(T_{F,W}\right)\gamma_{w}$$

(30) $$Y_{w}=1-0.5X_{\mathrm{NaCl}}-10X_{\mathrm{NaCl}}{}^{2}$$

(31) $$X_{\mathrm{NaCl}}=\frac{W_{\mathrm{NaCl}}}{M_{\mathrm{NaCl}}}/\frac{W_{\mathrm{NaCl}}}{M_{\mathrm{NaCl}}}+\frac{W_{w}}{M_{w}}$$

(32) $$p_{V}{}^{S}\left(T\right)=23.1964-\frac{3816.44}{T-46.13}$$

The transmembrane mass transfer is the micro transfer of molecules in porous media which may be realized in three modes: Molecular diffusion, Knudsen diffusion and Poiseuille flow according to the Knudsen number *K*_n_ defined by Equation (33) [24]:

(33) $$K_{n}=\frac{\lambda }{d}$$

where *λ* (m) is the average free path of water vapor, and *d* (m) is the diameter of membrane pores. Due to the existence of insoluble air in the membrane pores, the average free path of air and water vapor molecules can be calculated by Equation (34):

(34) $$\lambda_{W-A}=\frac{\kappa_{B}T_{M}}{\pi {\left(\frac{\delta_{W}+\delta_{A}}{2}\right)}^{2}p\sqrt{1+\left(\frac{Mwt_{W}}{Mwt_{A}}\right)}}$$

where *k*_B_ is Boltzmann’s constant (1.381 × 10^−23^ J·mol^−1^·K^−1^), *T*_M_ is the average temperature of the two membrane sides; *δ*_A_ (2.641 × 10^−10^ m) and *δ*_W_ (3.711 × 10^−10^ m) are the collision diameters of air and water molecules respectively; *p* is absolute pressure (Pa); *Mwt*_W_ and *Mwt*_A_ are the molecular weights of water and air, respectively. Generally, only the Molecular diffusion and Knudsen diffusion are considered to calculate the mass transfer coefficient *K*_M_ when the total pressure of both sides are equal. Thus, *K*_M_ can be calculated by Equation (35), which includes the molecular diffusion and Knudsen diffusion.

(35) $$K_{M}={\left(\frac{1}{K_{1}}+\frac{1}{K_{2}}\right)}^{-1}$$

(36) $$K_{1}=\frac{2}{3}\frac{r\varepsilon }{\tau \delta }{\left(\frac{8M_{w}}{\pi RT_{M}}\right)}^{0.5}$$

(37) $$K_{2}=\frac{D_{\mathrm{Wa}}\varepsilon }{\tau \delta }\frac{PM_{w}}{RT_{M}}$$

The value of *D*_Wa_ can be obtained by the following empirical formula:

(38) $$D_{\mathrm{Wa}}=1.895\times 10^{-5}T^{2.072}$$

The transmembrane heat transfer in the DCMD module occurs following three different processes: (1) heat is first transferred from the main body of the feed fluid to the membrane surface on the feed channel through the thermal boundary layer near the membrane surface; (2) then, the heat is transferred to the membrane surface on the cooling channel through the membrane in the form of conduction and vaporization, which reduces the fluid temperature in the feed channel [24]; (3) the vaporized steam on the feed channel passes through the membrane and condenses in the permeate channel, increasing the fluid temperature of the permeate channel. Therefore, the transmembrane heat transfer takes place in the form of phase transition and conduction, and the heat transfer flux *q*_M_ (w·m^−2^) can be calculated by Equation (39):

(39) $$q_{M}=q_{H}+q_{C}=J_{M}\Delta H_{V}+\frac{\kappa_{M}}{\delta }\left(T_{F,W}-T_{P,W}\right)$$

where *q*_H_ (w·m^−2^) is the latent heat of vaporization through the membrane while *q*_C_ (w·m^−2^) is the conducted heat which is considered as heat loss. *δ* (m) is the thickness of the membrane, and the enthalpy of evaporation of water *ΔH*_V_ (kJ·kg^−1^) can be calculated by Equation (40).

(40) $$\Delta H_{V}=-0.001351T_{F,W}{}^{2}-1.4461T_{F,W}+2986.5$$

#### 3.3.2. Balance Equations of DCMD Module

For the DCMD module, the balance equations are as follows:

(41) $$\mathrm{Mass}:\;{\dot{m}}_{10}+{\dot{m}}_{19}={\dot{m}}_{8}+{\dot{m}}_{9}$$

(42) $$\mathrm{Energy}:{\dot{m}}_{10}h_{10}+{\dot{m}}_{19}h_{19}={\dot{m}}_{8}h_{8}+{\dot{m}}_{9}h_{9}$$

(43) $$\mathrm{Entropy}:\;{\dot{m}}_{10}s_{10}+{\dot{m}}_{19}s_{19}+{\dot{S}}_{\mathrm{gen},\mathrm{med}}={\dot{m}}_{8}s_{8}+{\dot{m}}_{9}s_{9}{}_{}^{}$$

(44) $$\mathrm{Exergy}:\;{\dot{m}}_{10}ex_{10}+{\dot{m}}_{19}ex_{19}={\dot{m}}_{8}ex_{8}+{\dot{m}}_{9}ex_{9}+Ex_{\mathrm{des},\mathrm{med}}$$

The flow rate of each feed channel can be calculated as follows.

(45) Q=Sv

where *S* (m^2^) and *v* (m·s^−1^) are the cross-section area and fluid speed of the feed channel. Thus, the number of the DCMD modules and the freshwater production rate can be derived as (46) and (47), respectively.

(46) $$N=\frac{{\dot{m}}_{19}}{Q}$$

(47) $${\dot{m}}_{d}=JN$$

The freshwater conversion rate *α* is defined as follows, indicating the conversion ratio of the freshwater from the seawater through desalination.

(48) $$\alpha =\frac{{\dot{m}}_{d}}{{\dot{m}}_{19}}$$

### 3.4. Thermodynamic Performance Evaluation

The thermal efficiency *η* and exergy efficiency *φ* of the power and water generation of the system can generally be written as:

(49) $$\eta =\frac{\mathrm{energy}\;\mathrm{in}\;\mathrm{product}\;\mathrm{output}}{\mathrm{total}\;\mathrm{energy}\;\mathrm{inputs}}\times 100\%$$

(50) $$\varphi =\frac{\mathrm{exergy}\;\mathrm{in}\;\mathrm{product}\;\mathrm{output}}{\mathrm{total}\;\mathrm{exergy}\;\mathrm{inputs}}\times 100\%$$

The performance of the system such as the thermal efficiency and exergy efficiency of the PGC, the WPC, and the integrated OTEC system are analyzed, with the corresponding calculating equations shown in the Appendix A.

## 4. Results and Discussions

### 4.1. Power Generation Sub-Cycle and Exergy Analysis

Bernardoni et al. [25] stated that ammonia was the most suitable working fluid for the circulation process under the condition of 28 °C for warm seawater and 4 °C for cold seawater. Borji et al. [26] found that during the operation of the turbine, once liquid appears in the machine, droplets were thrown towards the outer edge of the impeller and vaporized sharply due to the high rotating speed of the working wheel in the rotor section, which made the clearance pressure fluctuate greatly and caused turbine vibration. Meanwhile, the generated high-speed droplets impact blades and the outlet of the guide vane, causing serious damage and accelerating blade breakage. The main reasons for liquid production are the low temperature of intake steam and its super-cooling caused by the high velocity. Therefore, the liquefaction of working fluid should be avoided as far as possible during the operation of turbine to maintain the stable operation state of the turbine.

**Figure 2:**
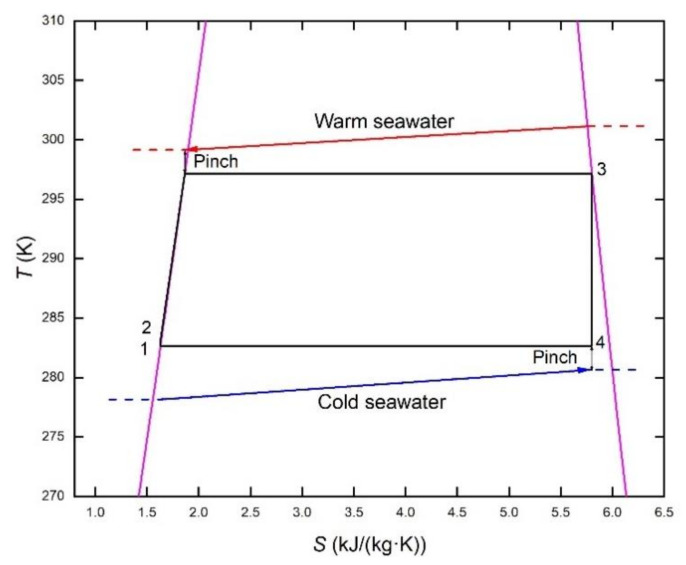
*T*-*s* diagram of dry-steam working fluid.

**Figure 3:**
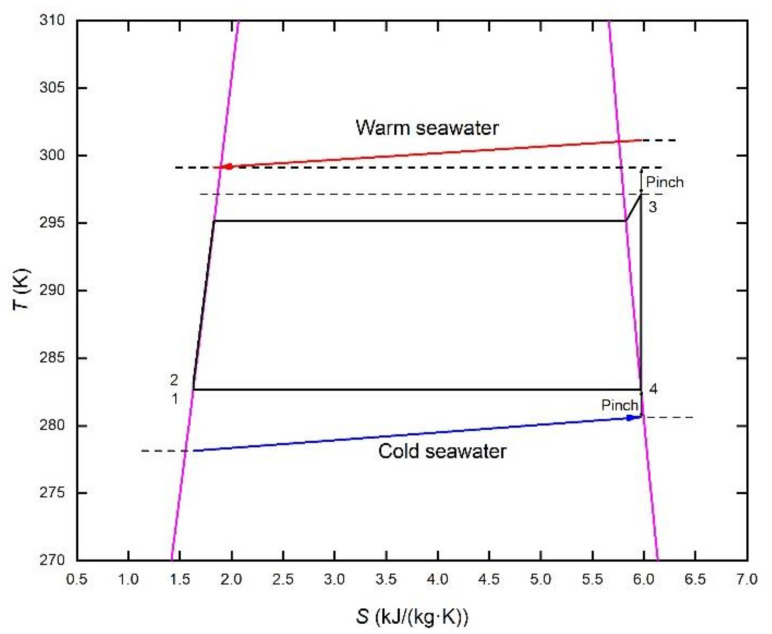
*T*-*s* diagram of dry-steam working fluid.

Figure 2 and Figure 3 show two organic Rankine cycles using ammonia as the working fluid under the condition of OTE. The difference is whether droplets occur during the expansion of ammonia steam in the turbine, i.e., whether the dry steam changes into the wet steam in the turbine. If the wet steam is allowed as the organic Rankine cycle shown in Figure 2, a larger temperature difference can be utilized. As shown in Figure 3, to maintain the dry-steam state in the turbine, the evaporating temperature is reduced from 24 °C to 22 °C with superheating of 2 °C to increase the relevant inlet temperature of the turbine avoiding droplet formation in the turbine impeller. Although part of the thermal efficiency is lost, the safe and stable operation of the turbine is guaranteed. The specific parameter settings for both cycles are shown in Table 1.

**Figure 4:**
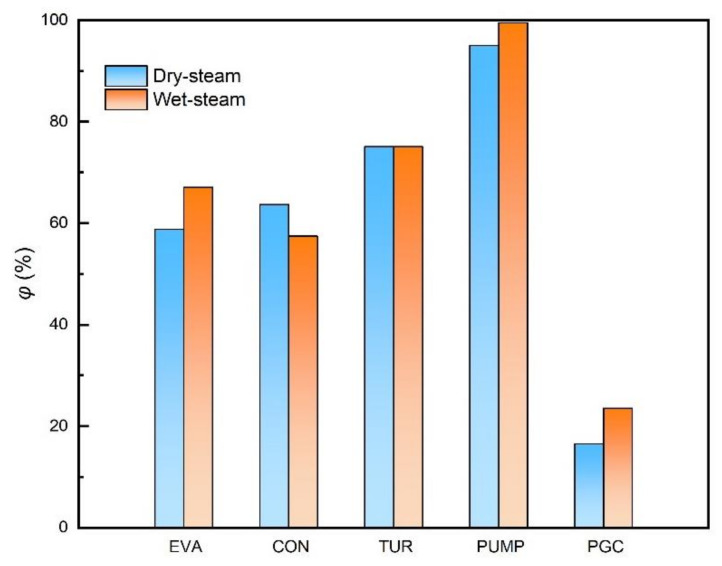
Exergy efficiency of the power generation sub-cycle (PGC) and key components.

Exergy efficiency is an important parameter to evaluate the energy conversion and utilization degree. Based on the cycles with working fluid of the above two states (“Wet steam” and “Dry steam”), the exergy efficiency of the PGC and the key components are discussed respectively, and the results are shown in Figure 4. The exergy efficiencies of the turbine are similar under the two working fluid state, the evaporator in the cycle with “Wet steam” working fluid performed better for the improvement of heat utilization, and thus, the exergy efficiency is higher. Similarly, the overall exergy efficiency of the PGC is higher for the “Wet steam” working fluid.

### 4.2. Water Production Sub-Cycle and Fresh Water Production

#### 4.2.1. Model Verification

**Figure 5:**
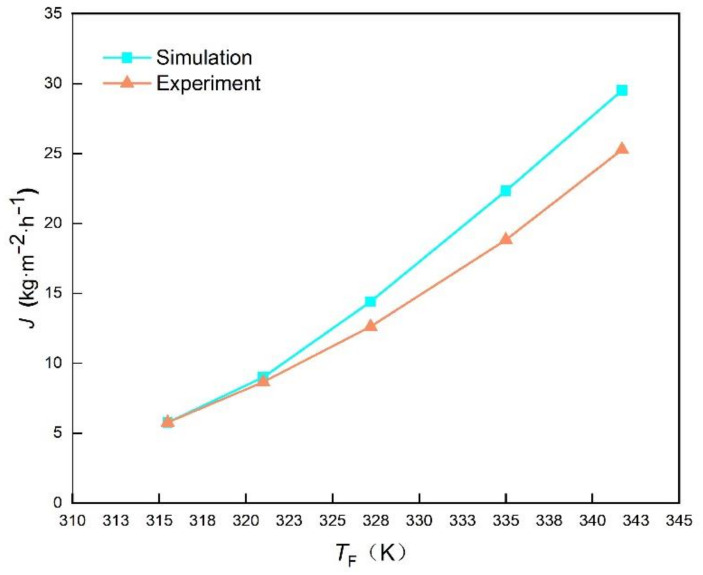
Comparison of experimental value and simulated value.

The WPC is based on the DCMD process, and the physical model of DCMD transmembrane transfer is embedded in the CFD software to predict the freshwater production performance of the WPC. The constructed CFD model is verified by comparing the simulation results with the experimental data from Hwang et al. [27] and Yun et al. [28] under the same structure and operation parameters. The feed temperature ranges from 309 K to 341 K, the permeate temperature is 292.7 K, the flow rate in both channels are 0.145 m·s^-1^, and the pressure is the standard atmospheric pressure. Figure 5 shows a comparison of simulated permeate flux with experimental data in literature. The simulated values match with the experimental values very well, and the permeate flux increases with the feed temperature, due to the increase of the saturated vapor pressure in the feed channel. Figure 5 also shows that the constructed model is better fitted with the experiment under the lower temperature conditions, indicating its potential to predict the DCMD permeate flux under the OTEC temperature condition.

*v*_F_ = *v*_P_ = 0.145 m·s^−1^, *p*_F_ = *p*_P_ = *p*_0_, *T*_P_ = 292.7 K,*W*_NaCl_ = 0

#### 4.2.2. CFD Prediction of Water Production Performance

**Figure 6:**
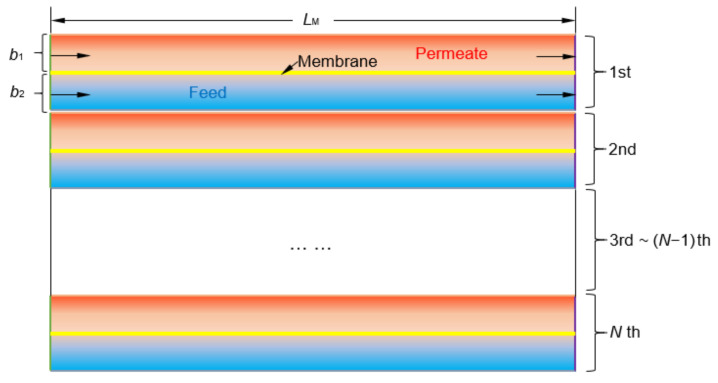
The membrane distillation system.

As shown in Figure 6, the DCMD system is composed of several plate DCMD models in parallel. The drained warm seawater from the PGC enters into the DCMD feed channel as the feed flow and the cycled fresh water cooled by the drained cold seawater from the PGC enters into the DCMD permeate channel as the permeate flow.

The temperature of both the drained warm and cold seawater will be affected by the working fluid state of the PGC as shown in Table 1. The inlet temperature of seawater in the feed channel is set according to the Table 1, the inlet temperature of cycled fresh water in the permeate channel is obtained from the heat transfer with the cold seawater, and the outlet temperature of the feed and permeate channel are calculated in the CFD model, the values of which are shown in Table 2.

**Figure 7:**
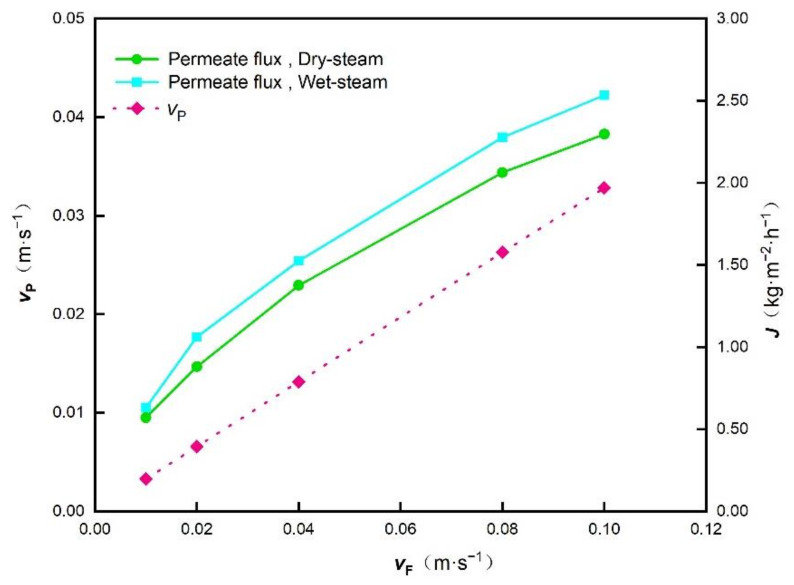
The influence of the feed channel velocity *v*_F_ on the permeate channel velocity *v*_p_ permeate flux *J*.

With constant seawater flow rates and channel cross-sectional area, when the speed in the feed channel changes, the number of modules and the speed in the permeate channel will change accordingly. The verified CFD model is used to predict the water production rate of the WPC, and the relationships between feed/permeate speed and permeate flux are shown in Figure 7. The speed in the permeate channel increases with the speed in the feed channel linearly. The increase of the feed and permeate speed results in the increase of the permeate flux. However, under the same seawater flow rate, the cross-sectional area of channel, and the membrane area of each module, the total membrane area will decrease with the speed. Thus, the speed increase will promote the permeate flux, but not the water yields necessarily.

**Figure 8:**
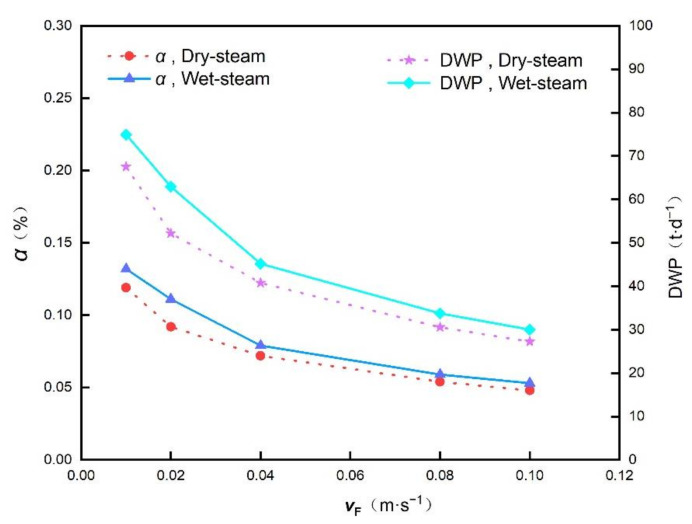
Influence of the feed channel velocity *v*_F_ on the water conversion rate *α* and daily water production (DWP).

In fact, as shown in Figure 8, as the feed channel speed increases, both the water conversion rate *α* and the daily water production rate (DWP) decrease. In pursuit of a higher conversion rate of the produced water, the speed in the feed channel is set to be 0.014 m·s^−1^ and that of the permeate channel is 0.0029 m·s^−1^ in this study. In practice, if there is space or membrane area limitation, the feed speed could increase to a proper extent to decrease the number of modules and the membrane area, and meanwhile, some loss of water production rate will be unavoidable.

**Figure 9:**
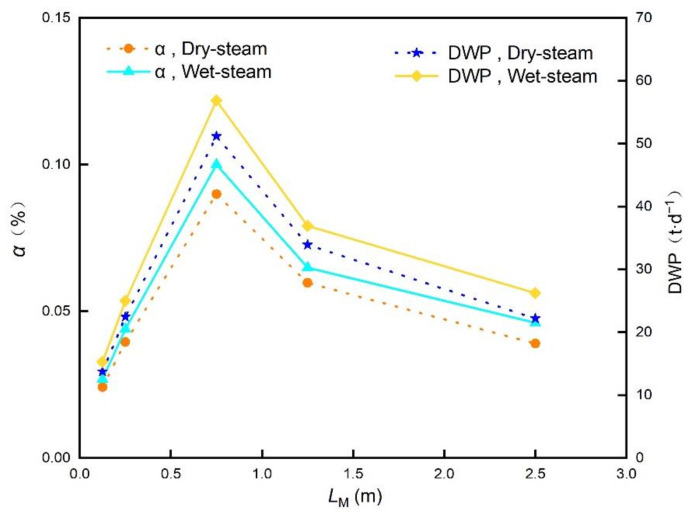
Influence of channel lengths *L*_M_ on the water conversion rate *α* and daily water production DWP.

In addition to the speed of the channels, the channel length *L*_M_ also has an effect on the permeate flux, indirectly affecting the water conversion rate (*α*) and daily water production (DWP). In this study, the investigated channel length ranges from 0.125 m to 2.5 m. Figure 9 shows the influence of channel length *L*_M_ on the *α* and DWP. The water production gradually increases with the channel length *L*_M_, in the beginning, with the maximum obtained when *L*_M_ =0.75 m, then rapidly decreases. Although the membrane area increases with the channel length, the heat loss also increases simultaneously which cause the decrease of the permeate flux. Thus, an optimal channel length exists inevitably under the combined action of the membrane area increase and the permeate flux decrease. In our study, this optimal value is 0.75 m, at which the rate of water conversion reaches the maximum of 0.1% (Wet steam), and the daily water production can reach 58.874 t/d (Wet steam) while the power output of PGC is 100 kW.

### 4.3. Analysis of the Integrated System

#### 4.3.1. Energy and Exergy Efficiency

Table 3 shows the exergy destruction rate and exergy destruction rate ratio of the integrated cycle and key components. The two working fluid states, i.e., the wet steam and dry steam, are both considered, with the output work of the ammonia heat engine cycle being 100 kW. The result shows that in the integrated system, the highest exergy destruction rate occurs in the evaporator, the condenser is the next and the lowest exergy destruction rate occurs in the working fluid pump. The dry-steam state causes larger exergy destruction than the wet-steam state, but the former is conducive to the protection of the turbine and maintain the stable operation state.

**Figure 10:**
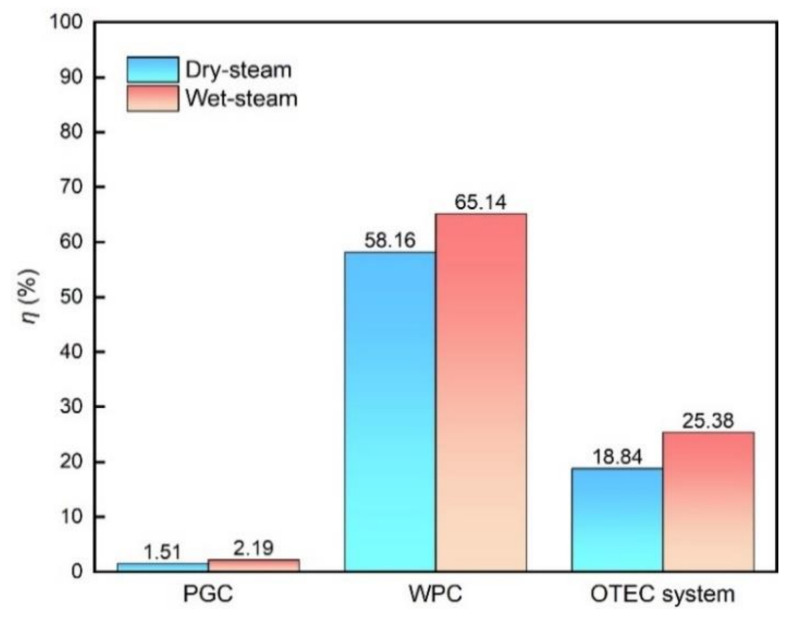
Thermal Efficiency of each cycle and the integrated system.

**Figure 11:**
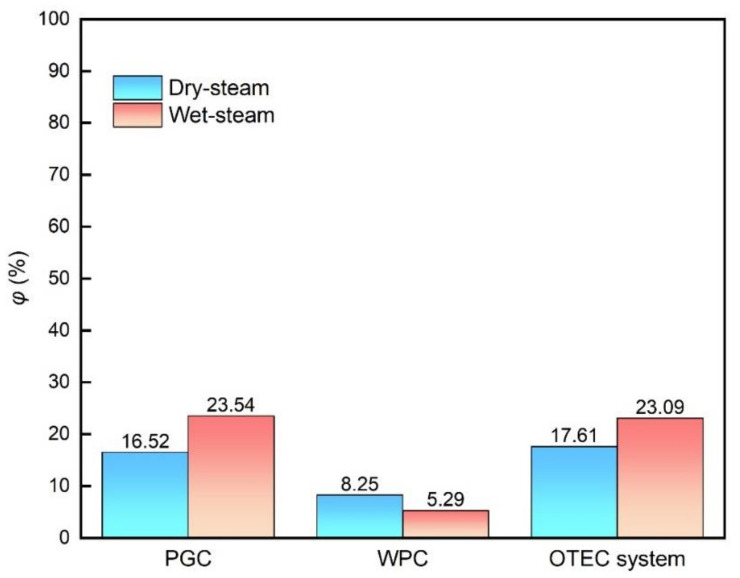
Exergy efficiency of each cycle and the integrated system.

In Figure 10 and Figure 11, the thermal efficiency and exergy efficiency charts for the PGC, the WPC and the integrated OTEC system are shown, respectively. Compared with the pure power generation cycle PGC, the efficiencies of the integrated system are promoted remarkably by coupling the WPC regardless of the state of the ammonia working fluid. Taking the results of the “Wet steam” working fluid system as an example, the thermal efficiency of the PGC is only 2.19%, which is greatly promoted after being combined with the WPC to 25.38%, and meanwhile, the exergy efficiency varies little, indicating that the OTE is utilized more effectively in the integrated system by recycling the drained warm and cold seawater from the PGC. After coupling with the DCMD, the residual thermal and cooling energy drained from the OTEC power generation cycle are reused to obtain freshwater, which increases the total output of the system, resulting in the remarkable increase of the thermal efficiency of the integrated system. In practice, there will be energy loss in the DCMD cycle, and the thermal efficiency of the integrated cycle will be deceased to an extent comparing to the above theoretical efficiency calculation. Still, the output efficiency of the integrated OTEC system is much higher than that of the conventional OTEC power generation system, and the proposed integrated system offers a good choice for the OTEC plant in the future.

#### 4.3.2. Economic Benefits

The OTE is a kind of clean ocean energy with lots of advantages mentioned in the beginning, indicating its great potential to supply the power and fresh water simultaneously for the tropical islands and coastal regions. However, whether the OTEC plant can be put into application is not only depending on the conversion technology but also the economic benefits. In this section, the economic benefits of the proposed OTEC integrated system are analyzed based on its output value in terms of typical demand markets, i.e., the sale benefits from electricity generation and water production, and other factors affecting the economic benefits like cost, operation, environment, etc., are not included. The electricity and water prices of 14 islands or coastal regions which suited for the OTEC exploration are adopted for the investigation. The price data are cited from [29] and shown in Table 4. The electricity and water prices differ a lot from regions. The average electricity price among 14 selected regions is $0.197/kWh and in Jamaica the electricity is the most expensive, with the price of USD $0.295/kWh. The average water price is USD 0.85/m^3^, and in Hawaii, the water is the most expensive, with the price of USD 2.23/m^3^.

**Figure 12:**
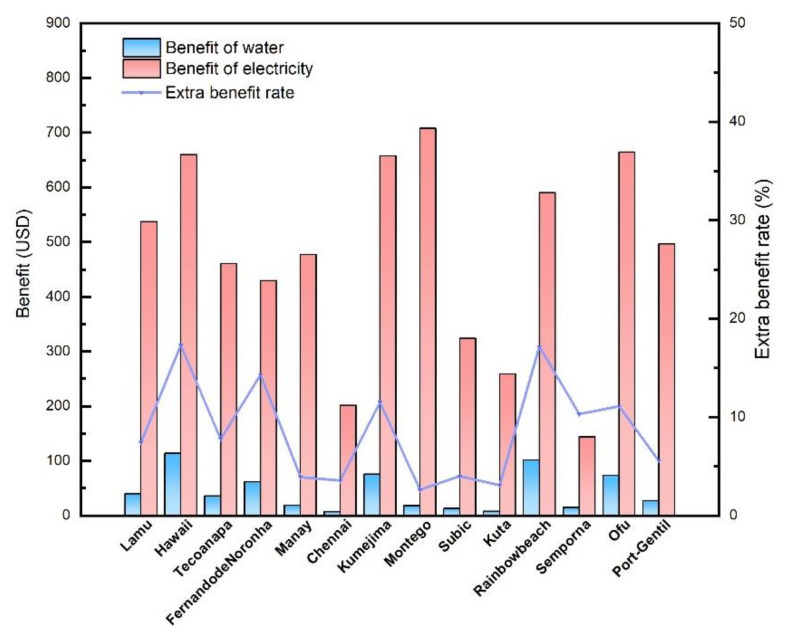
Daily benefit of water and electricity in different regions (Dry steam).

**Figure 13:**
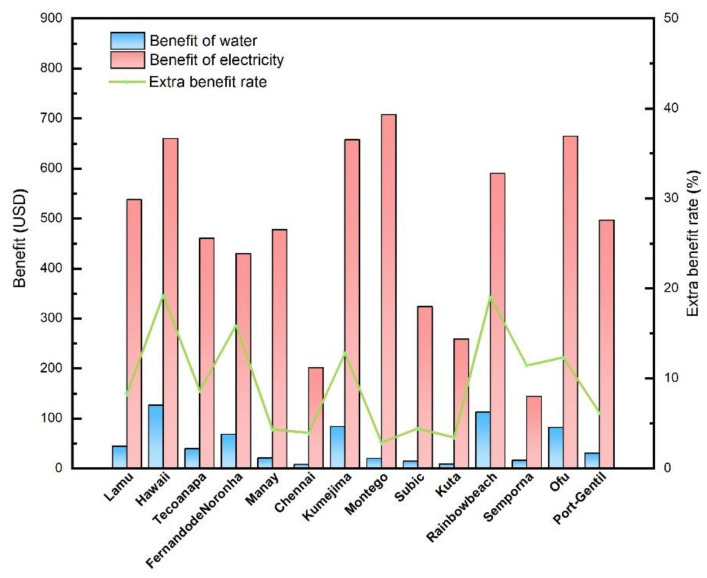
Daily benefit of water and electricity in different regions (Wet steam).

According to the price data and the output prediction, the daily profit of the proposed OTEC integrated system in selected regions are calculated and shown in Figure 12 and Figure 13, with the PGC working fluid of “Dry steam” and “Wet steam”, respectively. For all the regions, the daily profit of the “Wet steam” system is better than that of the “Dry steam” system. Although the profit of electricity is much remarkable than that of water, the water production offers extra benefit for the system since the WPC recycles the drained seawater from the upstream PGC. The extra benefit ratio can be calculated by the Equation (51). The average extra benefit ratio among the selected regions is around 9.49%. In particular, in Hawaii, this ratio approaches to nearly 20%, indicating an attractive promotion of the OTEC application. Meanwhile, the combined electricity and water supply characteristic of the proposed integrated system makes itself more suitable to solve the power and water shortage of the tropical islands and costly regions.

(51) $$\mathrm{Extra}\;\mathrm{benefit}=\frac{\mathrm{Benefit}\;\mathrm{of}\;\mathrm{water}}{\mathrm{Benefit}\;\mathrm{of}\;\mathrm{electricity}}\times 100\%$$

## 5. Conclusions

In this study, a novel ocean thermal energy driven system for sustainable power and fresh water supply coupling a power generation sub-cycle (PGC) and a water production sub-cycle (WPC) is proposed. The PGC is in the upstream of the system and adopts the simple organic Rainkine cycle for the power generation and the WPC is in the downstream of the system and adopts DCMD process to desalinate water. By thermal dynamic analysis and CFD simulation, the performance of the proposed system was investigated. After coupling with the DCMD desalination, the thermal efficiency of the OTEC system can be greatly improved from 2.19% to 25.38% under the optimal conditions. For a 100 kW OTEC power generation cycle, the water production rate approached 58.874 t/d. The water conversion rate is similar to the low-pressure flash process but the equipment volume can be compacted effectively; and based on the price data of 14 tropical coastal regions which are suitable for the application of OTEC and the resulted electricity and water generation data, the economic analysis according to the electricity and water sale was carried out. The economic profit can be improved by extra water production, especially in the Hawaii and Rainbow Beach, by nearly 20%.

Through the coupling of organic Rankine cycle and DCMD, the joint output of OTEC power generation and water production can be realized. The integrated system can not only improve the thermal efficiency of the OTEC plant, but also greatly increase the return on investment and the economic benefits. Meanwhile, the proposed integrated OTEC system is suited to solve the power and water shortage faced by most tropical islands and coastal regions, promoting the formation of a green, environmentally friendly and low-carbon lifestyle.

## Figures and Tables

**Table 1:** The ocean thermal energy conversion (OTEC) system configurations.

Working Fluid	Parameters	Dry Steam	Wet Steam
Ammonia	Evaporating temperature, °C	22	24
	Evaporating pressure, bar	7.3	8
	Temperature of evaporator outlet, °C	24	24
	Condensing temperature, °C	9.5	9.5
	Condensing pressure, bar	5.99	5.99
	Temperature of condenser outlet, °C	9.5	9.5
	Mass flow rate of working fluid, kg/s	5.131	3.544
Seawater	Temperature of warm seawater inlet, °C	28	28
	Temperature of cold seawater inlet, °C	5	5
	Mass flow rate of warm seawater, kg/s	658.263	658.263
	Temperature drop of warm seawater, °C	2.22	1.53
	Mass flow rate of cold seawater, kg/s	523.413	523.413
	Temperature rise of cold seawater, °C	2.75	1.88
	Power generation of turbine, kW	100	100
	Working fluid pump power, kW	1.361	1.344

**Table 2:** The temperature of Direct contact membrane distillation (DCMD) system.

Parameters	Dry Steam	Wet Steam
The inlet temperature of permeate channel, °C	9.7	8.8
The outlet temperature of permeate channel, °C	16.14	14.98
The inlet temperature of feed channel, °C	25.8	26.47
The outlet temperature of feed channel, °C	24.99	25.58

**Table 3:** Exergy destruction of components.

Components	Exergy Destruction Rate (kW)		Exergy Destruction Ratio (%)	
	Dry Steam	Wet Steam	Dry Steam	Wet Steam
Evaporator	249.583	139.773	50.11	41.24
Condenser	81.357	65.201	16.34	19.24
Turbine	33.073	33.131	6.64	9.77
WF pump	2.656	2.682	0.53	0.79
DCMD	70.805	40.831	14.22	12.05
Heat exchanger	60.571	57.333	12.16	16.91
Total	498.045	338.952	100	100

**Table 4:** Tropical coastal regions suitable for application of OTEC system.

Regions	Electricity Price (USD/kWh)	Water Price (USD/m^3^)
Lamu (Kenya)	0.224	0.78
Hawaii (USA)	0.275	2.23
Tecoanapa (Mexico)	0.192	0.7
FernandodeNoronha (Brazil)	0.179	1.2
Manay (Philippine)	0.199	0.365
Chennai (India)	0.084	0.14
Kumejima (Japan)	0.274	1.48
Montego (Jamaica)	0.295	0.36
Subic (Fiji)	0.135	0.253
Kuta (Indonesia)	0.108	0.157
Rainbowbeach (Australia)	0.246	1.98
Semporna (Malasysia)	0.06	0.29
Ofu (Samoa)	0.277	1.44
Port-Gentil (Gabon)	0.207	0.54

## Data Availability

The authors declare that all data supporting the findings of this study are available within the article.

## Nomenclature

Q	Heat transfer rate (kW)
W	Work rate (kW)
S	Entropy (kW)
Ex	Exergy (kW)
m	Mass flow rate (kg·s^−1^)
h	Specific enthalpy (kJ·kg^−1^)
s	Specific entropy (kJ·kg^−1^·K^−1^)
J	Permeate flux (kg·m^−2^·h^−1^)
T	Temperature (℃,K)
V	Flow rate (m^3^·s^−1^)
A	Cross-section area (m^2^)
v	Velocity (m·s^−1^)
N	Number of modules
ΔE	The change of exergy (kW)
h_r_ c	The latent heat of vaporization (kJ·kg^−1^) Specific heat capacity (kJ·kg^−1^·K^−1^)
Greek letters	
η	Thermal Efficiency (%)
φ	Exergy Efficiency (%)
α	Conversion rate (%)
τ	Efficiency of turbine (%)
θ	Efficiency of pump (%)
Subscripts	
Con	Condenser
Eva	Evaporator
WF	Work fluid
WCR	Daily water production
des	Destruction
gen	Generation
t	Turbine
in	Inlet
out	Output
ph	Physics
w	Work
F	Feed channel
P	Permeate channel
h	Hot
c	Cold
d	Desalination
b	Breadth of modules

## Appendix A

**Table A1:** Thermal and exergy efficiency of each cycle.

Cycle	Energy Efficiency	Exergy Efficiency
Integrated OTEC System	$\eta_{\mathrm{System}}=\frac{W_{\mathrm{out}}+\alpha m_{19}h_{r}}{cm_{19}\Delta t}$	$\varphi_{\mathrm{System}}=\frac{W_{\mathrm{out}}+\alpha m_{19}ex_{19}}{Ex_{\mathrm{System}}}$
WPC	$\eta_{\mathrm{WPC}}=\frac{\alpha m_{19}h_{r}}{Q_{\mathrm{WPC}}}$	$\varphi_{\mathrm{WPC}}=\frac{\alpha m_{19}ex_{19}}{Ex_{\mathrm{WPC}}}$
PGC	$\eta_{\mathrm{PGC}}=\frac{W_{\mathrm{out}}}{Q_{{}_{\mathrm{PGC}}}}$	$\varphi_{\mathrm{PGC}}=\frac{W_{\mathrm{out}}}{Ex_{\mathrm{PGC}}}$

where, *h_r_* is the latent heat of vaporization, kJ/kg.

**Table A2:** Exergy efficiency equations of major components of the PGC.

System Components	Exergy Efficiency Equations
Evaporator Condenser	$\varphi_{E}=\frac{\Delta E_{c}}{-\Delta E_{h}}$ $\varphi_{C}=\frac{\Delta E_{c}}{-\Delta E_{h}}$
Turbine	$\varphi_{T}=W_{t}/m_{3}(ex_{4}-ex_{3})$
WF pump	$\varphi_{P}=P_{\mathrm{WF}}/m_{5}(ex_{6}-ex_{5})$

where Δ*E*_c_ is the change in exergy of the cold fluid, and Δ*E*_h_ is the change in exergy of the hot fluid.
